# Evaluation and Use of Registry Data in a GIS Analysis of Diabetes

**DOI:** 10.3934/publichealth.2015.3.318

**Published:** 2015-07-23

**Authors:** Mungrue Kameel, Sankar Steven, Kamalodeen Aleem, Lalchansingh Dayna, Ramnarace Demeytri, Samodee Shanala, Sookhan Craig, Sookar Navin, Sooknanan Kristal, St.George Leah, Suruj Deonath

**Affiliations:** 1 University of the West Indies, Faculty of Medical Sciences, Department of Paraclinical Sciences, Public Health and Primary Care Unit, India

**Keywords:** type 2 diabetes mellitus, GIS, spatial epidemiology

## Abstract

**Objectives:**

to evaluate registry data routinely collected by the Chronic Disease Electronic Management System (CDEMS) in the monitoring of type 2 diabetes mellitus (T2DM) in the Eastern half of the island and use the data to describe the spatial epidemiological patterns of T2DM.

**Design and Method:**

The starting point was access and retrival of all exsisting data on the diabetes registry. This data was subsequently validated using handwritten medical records. Several clinical indicators were selected to evaluate the registry. The address of each patient was extracted and georeferenced using ArcGIS 10.0 and several maps were created.

**Results:**

The registry had data for thirteen (13) out of the sixteen (16) health facilities. We found that less than 15 percent of all patients actually had diabetic indicator tests done according to World Health Organization (WHO) standards. The overall prevalence of T2DM was 20.8 per 1000 population. The highest prevalence of diabetes occurred at the northeastern tip of the island. In addition 57.58% of patients with T2DM resided inland and 40.75% of patients residing on the coastal areas.

**Conclusions:**

In conclusion, we provide evidence that the data collected by the diabetes registry although lacking in many areas was adequate for spatial epidemiological analysis.

## 1. Introduction

Attaining targets set by evidence based international guidelines in patients with Type 2 Diabetes (T2DM) remains a challenge particularly in the developing world. Bodenheimer and collegues [1] developed a chronic care model as a conceptual framework for planning interventions to change current chronic disease care. This model describes six key components for managing patients with a chronic illness in a primary care setting. The six components consists of (1) clinical information systems, (2) self-management support, (3) delivery system redesign, (4) decision support, (5) health care organization, and (6) community resources. This model has been validated for T2DM. [2]–[4] Furthermore the chronic care model emphasizes information technology as a critical component in the delivery of optimal care. The model recommends the creation and maintenance of a registry. In its simplest form, a registry is a list of patients which captures data on a particular disease in a defined setting [5],[6]. Registries should flag patients who are not meeting recommended targets. In Trinidad particularly glycosylated haemoglobin levels (HbA1c) and microalbuminuria in T2DM are key examples. In addition registries can be used to (1) generate follow-up reports on clinical course and outcomes associated with interventions (e.g. progress with the healing of a septic lesion on the foot, may avert an amputation); (2) to identify patients who are noncompliant, which may lead to a hospital admission; (3) to establish a communication link between the health facility and the patient; and (5) to identify high-risk patients [7]–[10]. Several studies have reported that registries can improve clinical processes and outcomes for patients with T2DM [11]–[14] and improve the quality of care [15]–[19].

Another important use of registry data is for mapping and spatial analysis. Few studies have used diabetes registry data for this purpose [20]–[22]. In fact this is the first study of its kind in Trinidad. Maps are elegant surveillance tools that can identify areas with the largest disease burden and provide insight into the underlying epidemiology of diabetes useful in guiding appropriate interventions.

The aim of this study therefore is to use registry data collected by the Chronic Disease Electronic Management System (CDEMS) in the monitoring of T2DM in the Eastern half of the island to describe the spatial epidemiological patterns of T2DM.

## 2. Methods

In Trinidad and Tobago, there is a two-tiered system of delivery of health care, which consists of a public health care system provided by the state is free to all clients and a private health care system based on a fee for service model. The population of Trinidad and Tobago is approximately 1.3 million while the population of the Eastern Region is 120,000 [23]. The population consists of two major diaspora South Asians (SA) and Africans representing 37.6% and 36.3% of the population respectively, both similarly represented in this region. The public health care system consists of four administrative regional health authorities (RHA). Each RHA is responsible for the delivery of all public health care services. The Eastern RHA (ERHA) delivers health care to approximately one third of the island. It is responsible for one (1) tertiary health facility and sixteen (16) primary care facilities (PCF). Among the services provided by the sixteen Primary Health Care Centres is the provision of care for patients with chronic diseases.

CDEMS is a public domain Microsoft access database developed by the Washington State Diabetes Prevention and Control Programme (DPCP) in 2002 [24]. It is designed to store and retrive patients' demographic data, visit dates, clinical characteristics, medications, laboratory reports and clinical notes in several data layers. The ERHA is the only health authority that has initiated a diabetic registry using CDEMS in an attempt to improve diabetic care. CDEMS was introduced in 2007 in all 16 PCF. The physician hand written medical record is converted by specially trained operators to convert this information into an electronic database. Hence the date entered into CDEMS was accessed and subsequently validated from the hand written medical records. The key indicators selected for monitoring diabetes care were adapted from a review of guidelines published by the International Diabetes Federation [25]. We examined and selected only appropriate key indicators such as annual foot examination, annual eye examination, annual nephropathy screen (urea & creatine ratio, 24 hr urine collection), annual LDL cholesterol, blood pressure, HbA1c, BMI, smoking and alcohol consumption. These key indicators were used to audit the proportion of people who attained these targets. Results such as foot examination, eye examination or nephropathy screen would be reported as a proportion [n/N (%)], HbA1c as < 7% or > 7%, annual triglyceride level, annual HDL cholesterol (> 50 mg/dL), annual LDL (< 100 mg/dL), total cholesterol (< 200 mg/dL) and blood pressure (< 130/80mm/Hg) measurements. For the purposes of this study we used The World Health Organisation (WHO) criteria for the diagnosis of T2DM i.e., (1) a fasting blood sugar ≥ 126 mg/dL (2) a 2 hour post prandial of ≥ 200mg/dL following a 75 g glucose load and (3) whether patients are currently receiving any combination of lifestyle intervention, oral anti-diabetic drugs or insulin therapy [26].

Addresses was collected for the purpose of mapping T2DM in the region using Arc GIS version 10.0 Address data was subsequently geo-coded using street data. The coordinates of each of the sixteen Health Centers were determined using a mobile Global Positioning System (GPS) device, which was subsequently merged with the street data using Arc GIS. Descriptive maps of the data were made using colored, topographic, high-resolution, 3-Dimensional map of Trinidad, with the exact location of each Health Centre clearly identified. An overlay technique was employed to illustrate the distribution of patients with T2DM in Eastern Trinidad on the same map. Population data for the respective areas was obtained from the Central Statistical Office. Data was then used in the calculation of prevalence rate. Ethical approval for the study was obtained from The University of West Indies Ethics committee.

## 3. Results

A total of 1930 patients with T2DM were entered into the registry. This represented 13 (81.2%) of the 16 PCF, although all facilities were equipped with both the software and hardware capabilities to collect data. In the three PCF without registry data we collected an 576-patients, which was combined with the 1930 patients in the registry. The total number of patients therefore available for analysis was 2506. However not all variables had 100% values entered either in the medical records or the registry. The average age in the combined databases was 61 years. The largest percentage (27.8%) of patients was in the age range 60–69 years. Although T2DM predominantly manifests in the fourth decade of life, 119(6.2%) patients were presenting in the age group less than 40 years, Table 1.

There were twice as many females (1205, 62.8%) than males (713, 37.2%) with a f:m ratio of approximately 2:1. The population of Trinidad consists of two major diaspora, South Asians (SA) and Africans both representing approximately 35% of the population. T2DM was significantly higher (*p* ≤ 0.05) in SA (46.4%) than Africans (31.2%). BMI was calculated for only 125 (6.5%) patients because height was not unavailable for all patients. Using the WHO classification of obesity [27], 42.4% was clinically obese (BMI > 29 kg/m^2^). Similarly 638 (33.1%) patients did not have a blood pressure (BP) entered into the registry data. Of the 1328 patients with recorded BP, 53% did not achieve the target for blood pressure control (< 140/90 mmHg) according to the JNC VIII recommendations for hypertension [28], Table 1. While international guidelines recommend lipid profiles for all patients with T2DM, under 15% of patients had a lipid profile recorded. The majority (> 55%) of these patients did not meet the targets set by international guidelines for HDL cholesterol (> 50 mg/dL), LDL (< 100 mg/dL) and total cholesterol (< 200 mg/dL), Table 2 [29].

**Table 1.** Characteristics of patients, with T2DM.

Characteristic		N (%)
**Age**		
< 40		119(6.2)
40–49		278(14.8)
50–59		455(24.1)
60–69		525(27.8)
70–79		372(19.7)
80–89		119(6.1)
≥ 90		22(1.2)
Total		1890
**GENDER**		
Male		713(37.2)
Female		1205(62.8)
Total		1918
**ETHNICITY**		
E.I		896(46.4)
African		603(31.2)
Other		431(22.4)
Total		1930
**BMI (kg/m^2^)**		
< 25		43(34.4)
25–28		29(23.2)
29–34		36(28.8)
≥ 35		17(13.6)
Total		125
**BP (mmHg)**		
Systolic		
Diastolic		
≤ 120		
≤ 80		
121–139	81–89	195(14.2)
140–159	90–99	452(32.9)
≥ 160		374(27.2)
≥ 100		354(25.7)
**Total**		1375 (100)

**Table 2:** the Distribution of HDL, LDL, TG and Total Cholesterol recorded.

Test	N (%)
**HDL (mg/dL)**	
> 50	60(27.5)
<50	158(72.5)
**LDL (mg/dL)**	
>100	159(75)
<100	53(25)
TC (mg/dL)*	
>200	163(56.8)
<200	124(43.2)
**TRIG (mg/dL)†**	
>150	109(41.9)
<150	151(58.1)

* = total cholesterol, † = triglyceride

An annual nephropathy screen to identify patients who are at risk for diabetic nephropathy was unavailable. However at some stage in the course of the disease 276 (14.3%) patients had a creatinine performed and 200 (10.4%) a serum urea.

An important target in T2DM is maintaining an HbA1c below 7%, a good indicator of glycaemic control. It is important to identify that only 55 patients had a recorded HbA1c and the majority (78.2%) had values above 7%, Table 4.

Two important assessments the annual foot examinations and the annual eye examinations were not accommodated in the CDEMS database and therefore were unavailable for evaluation.

CDEMS can also be used to record current therapy. While the majority of patients were on oral agents 11.1% of patients were receiving insulin therapy table 5.

We obtained the address of each patient. This data was georeferenced using ArcGis 10.0 (ESRI) to create several maps. The specific analytical objectives were visualization and exploration to examine the spatial dimensions of the data. Disease counts were expressed as a function of the population size to provide estimates of the proportion of people with T2DM. The first map demonstrates the point distribution of the raw data to allow the appreciation of spatial patterns without the burden of technical details of analyses done to facilitate data display. It appears that more cases occurred in the northern half of the region as well as the coastal areas. Further analysis using a cartogram to representing the proportion of T2DM, is displayed in Figure 3. The choice of a cartogrm over a choropleth map was chosen (1) to reduce component polygons of the study region that are large and may dominate the display and lead to bias in interpretation, (2) to reduce the modifiable areal unit problem (MAUP) effect and (3) highly skewed distributions are difficult to display using a finite number of color shading scales. The region was therefore divided into population sizes approximately 5000–6000 population. However we used the Moran's Index (*I*) in ArcGIS to map the clustering of diabetes prevalence across communities. Moran's *I* ranges from −1.0, perfectly dispersed (e.g., a checkerboard pattern), to a +1.0, perfectly clustered. A *z* score and *P* value are generated as outputs along with Moran's *I*.

Diabetes prevalence in the ERHA at the community level ranged from 0.5 per 1000 to 129 per 1000 and was significantly clustered (Moran's *I* = 0.35; *z* = 540.2; *P* < .001). The highest cluster of diabetes prevalence occurred at the extreme northeastern peninsula of the island, table 6. The overall prevalence of T2DM was 20.8 per 1000. 13 out of the 16 health facilities (81.3%) had a prevalence of less than 60 per 1000.

**Table 3.** The distribution of patients who had a Nephropathy Screen.

Test	N (%)
Creatinine (mg/dL)	
> 1.4	24(8.7)
< 1.4	252(91.3)
Total	276
Urea (mg/dL)	
> 40	3(1.5)
< 40	197(98.5)
Total	200

**Table 4.** The proportion of Patients with an HbA1c ≤ 7% or > 7%.

HbA1c (%)	n(%)
> 7	43(78.2)
≤ 7	12(21.8)
Total	55

**Table 5.** Current Therapy classified as oral agents and insulin.

Treatment option	N (%)
Insulin	214(11.1)
Oral medication (OM)	850(44)
Insulin & OM	118(6.1)
Non-pharmacologica (only)	748(38.8)
Total	1930 (100)

**Table 6.** prevalence of T2DM by the population of the community.

Community	Patients with T2DM	Population	Prevalence of T2DM (x1000)	Community	Patients with T2DM	Population	Prevalence of T2DM (x1000)
Rio claro	347	11852	29	Toco	168	1302	129
Guaya	72	2303	31	Mathura	154	1297	119
Biche	172	4303	40	Coryal	90	1854	48
Mayaro	363	7666	47	Cumuto	227	4628	49
Cumana	83	1601	51	Manzanilla	310	5325	58
Grand Riviere	11	298	37	Sangre Grande	11	21791	0.5
Matelot	21	486	43	Valencia	266	7372	36
San Souci	24	612	39	Brother's Road	187	2285	82
Total		24818				45868	

**Figure 1.**
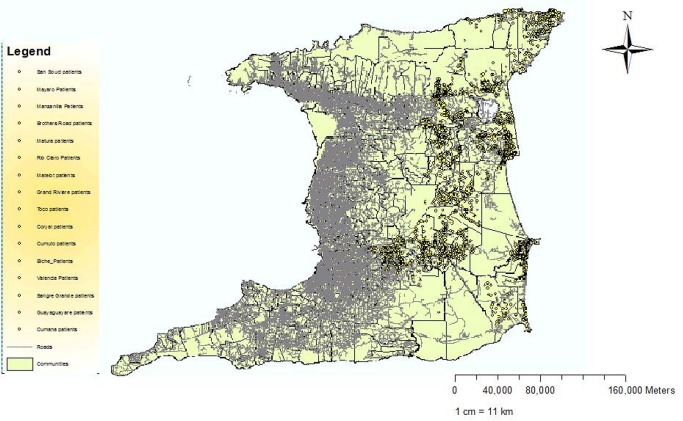
Point map showing the distribution of all cases of T2DM in the ERHA.

**Figure 2.**
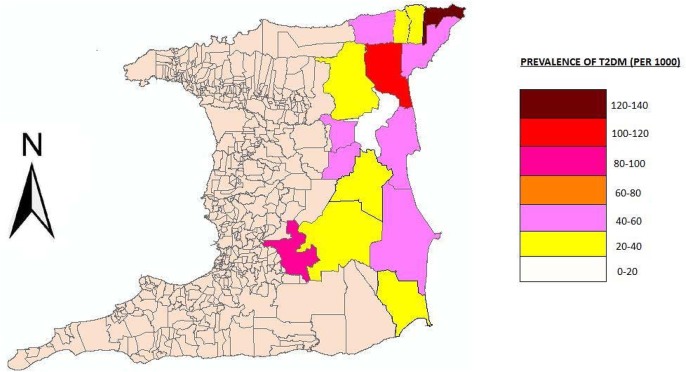
A cartogram of Prevalence of T2DM expressed as the number of cases per 1000, where the boundaries of each area represents population size.

We created a buffer map to compare the prevalence in coastal areas within land areas. Coastal areas were defined as a perimeter of 500m inland from the coast line, while inland areas was defined as the distance from the inner perimeter of the costal area to the boundary of the ERHA. Using these definitions two buffer zones were created and the prevalence of T2DM calculated for each and displayed as a choropleth map, Figure 4. The inland prevalence (P_i_ ) was 57.58 per 1000 which was significantly higher (*p* = 0.023), than the coastland prevalence (P_C_) of 40.75 per 1000.

Finally we calculated the distance between the patient's location and the health facility to determine the utility of each facility, Figure *5*. While most people (95%) attended their designated health facility, we found that 5% of patients who lived closest to the health facility sought medical attention elsewhere.

**Figure 3.**
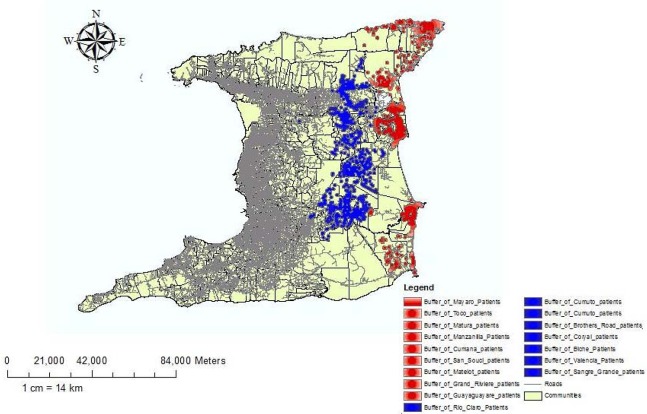
A Buffer Map comparing the Prevalence of T2DM in inland area and coastal areas expressed as the number of cases per 1000.

**Figure 4.**
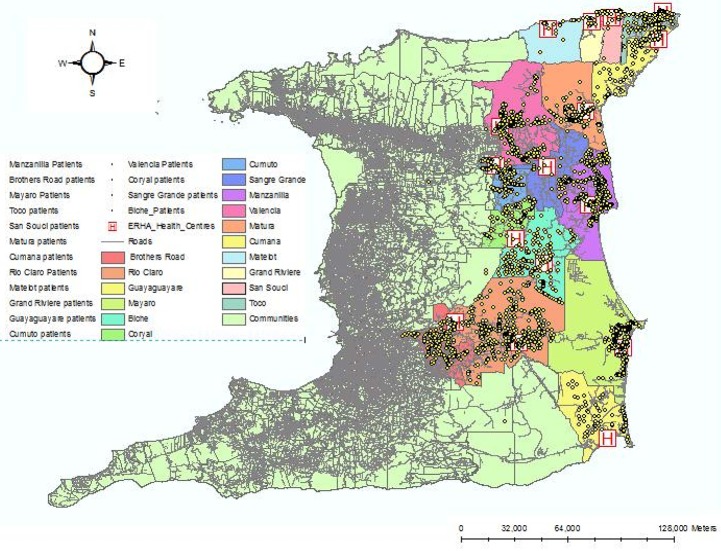
Showing T2DM Patient's Proximity to the Health Facilities.

## 4. Discussion

A chronic disease electronic registry was first implemented in Trinidad a small island developing country in 2007. We sought to assess the quality and extent of the data collected and its role in understanding the spatial epidemiology of T2DM.

The predominant age group affected by T2DM is the age group 60–69 years (27.8%) while the combined age groups 50–59 years and 60-69 years accounted for a half of all cases. An important finding was as much as 119 (6.2%) patients in the age group ≤ 40 years which included adolescents were currently receiving treatment for T2DM, a disease once thought to be a metabolic disorder exclusively of adulthood [30]. This may represent the increasing phenomenon of maturity onset diabetes in the Young (MODY) [31]. This trend in the occurrence of T2DM is not unique to our setting. For example, in Japan 80% of all new cases of diabetes in children and adolescents were diagnosed as T2DM [32]. Similar patterns have been reported in Taiwan ^28^, and to a less extent in Europe, U.K. and the U.S.A [33], [34].

Among the two major diaspora in the population T2DM was significantly higher in SA (46.4%) compared with Africans (31.2%), a finding first reported in 1961[34].and corroborated by Beckles and Miller in the St.James Cardiovascular Survey [35], and more recently by Chadee et al [36]. Forty-four years later we report similar findings. The f:m ratio approached 2:1 indicating that T2DM has strong gender disparity, a finding supported by other studies[37]. The implications of this finding are the impact T2DM is expected to have on the future health of women. Both microvascular and macrovasular complications i.e. diabetic retinopathy, end-stage renal disease, lower extremity amputations, myocardial infarction and stroke, particularly as life expectancy is higher in women (73 years) than men (67 years) places women at higher risk. This finding also underscores the urgent need to identify and target women at risk of developing T2DM for health promotion interventions to prevent the onset of T2DM, particularly in childbearing years where they are exposed to health care professionals. This is against a background in which Boda et al reported that this region has the largest geographical area and distance becomes a barrier to access health care requiring substantial driving times to reach these clinics [38].

Our findings demonstrate unequivocally that the current quality of data contained in the registry was poor. In the first instance no data was collected in three PCF. The software lacked the capability to record and track annual examinations of the feet and the eyes in patients with T2DM. Annual foot examinations can improve the early detection of diabetic neuropathy and vasculopathy, and prevent diabetic trauma, ulcers, gangrene and amputations by 25% [39]. A reduction by one-third or more in new blindness due to diabetes has been adopted as one of the key 5-year targets in the St Vincent declaration, fundoscopic examinations are effective in reducing the incidence of diabetic retinopathy by as much as 50% [40]. Further 10.4% and 14.3% of patients had a urea and creatinine recorded respectively. Trinidad has the highest number of patients currently receiving dialysis for end stage renal disease in the Caribbean, which is mainly due to diabetes and hypertension[41]. Thus reemphasizing the importance of monitoring renal function in T2DM.

Using the WHO criteria for obesity [42] (BMI > 29.9), 42.4% of patients were obese. Although only 125 patients i.e. less than 10% overall had available data to calculate BMI, the effect size is large to support the strong epidemiological evidence that links obesity and diabetes [43]–[46]. Dyslipidaemia is a common finding in T2DM to the extent that statin therapy is recommended for all patients with DM. We observed that only 50% of patients with T2DM had a lipid profile recorded. Further the majority of patients failed to meet the targets set by international guidelines for HDL (72.5%), LDL (75%), and total cholesterol (56.8%) [47].

An elevated blood pressure (>140/90 mmHg, JNC VIII) was recorded in 53% of all patients. In patients with diabetes, hypertension is more difficult to control as the optimal blood pressure goal is lower and more difficult to achieve [46]. In addition, Sowers and colleagues found that hypertension is approximately twice as frequent in patients with diabetes as compared to patients without [48]. Further Padilla and colleague showed that T2DM and hypertension is a common clinical scenario that can set off a vicious cycle of increasing renal damage, and increased cardiovascular morbidity and mortality [49]. Although the diabetes registry was designed to monitor patients with T2DM to improve clinical care and outcomes, this finding alone demonstrates it was not achieving these objectives. Further research along the lines proposed by Bodenheimer et al[50], and relevant to the developing world such as lack of resources, inadequate information technologies, physician resistance and inertia all need to be investigated to provide corrective strategies. On the other hand we were able to obtain specific locations of all patients with diabetes in the ERHA, which we used to create several maps. The point distribution of all cases is shown in Figure1, while Figure 2 is a cartogram of the prevalence of T2DM within the ERHA. An unusual finding was the highest prevalence (120–140 per 1000) of T2DM was located in the community living in the northeastern tip of the island. While we may speculate that many factors can account for this high prevalence of diabetes such as environmental genetic predisposition, lifestyle and socio-economic factors, we cannot offer an exact explanation. Nonetheless this finding demonstrates that spatial analysis can still be used for geographical targeting to improve detection and address diabetes risk.

The proportion of cases for inland areas (57.8%) was significantly (*p* < 0.05) higher than coastal areas 40.75% (Figure 3). This finding may be explained by environmental factors and socio-economic factors. Using the high cost of costal properties as a marker of socio-economic status residents of coastal areas are more likely to have higher disposable incomes and consequently seek private care rather than utilize public health care facilities. On the other hand environmental factors such as inhospitable terrain and poor infrastructure are more likely to attract residents of lower socio-economic status who are more likely to utilize public health care facilities. However an array of other factors such diet, physical activity, genetic and ethnic disparities and diabetes education may all act as contributors. Finally, Figure 4 shows the relationship between the point distribution of diabetes and the location of health facilities. Essentially, the closer patients lived in proximity to the health facility, the higher was the number of cases diagnosed, which is expected as the PCF is usually located in the center of the community. However, we found an interesting phenomenon in which approximately 5% of clients who live very close to health facilities did not attend those facilities but attended facilities further away. A possible explanation is a perception among patients that some health facilities offer better care than others.

The study has a number of methodological limitations. First, it has relied exclusively on data derived from administrative and clinical databases in order to estimate the DM prevalence rates. Since cases have not been individually verified, this approach could result in either an overestimate or underestimate of prevalence rates. Secondly it is possible that existing variability in health care access and diagnostic practices could influence the level of care delivered.

In conclusion, the objective of the paper was to show how data that is routinely collected by PCF could be used to gain an insight into local area occurrence of T2DM. Although this is a first exploratory analysis, the results reveal interesting issues worthy of further investigation. Moreover it raises questions on the need to better understand the powerful and predictable impact that place has on the health of populations.

## 5. Conclusion

In conclusion, the objective of the paper was to show how data that is routinely collected by PCF could be used to gain an insight into local area occurrence of T2DM. Although this is a first exploratory analysis, the results reveal interesting issues worthy of further investigation. Moreover it raises questions on the need to better understand the powerful and predictable impact that place has on the health of populations.

## References

[1] Bodenheimer T, Wagner EH, Grumbach K (2002). Improving primary care for patients with chronic illness. JAMA.

[2] Bodenheimer T, Wagner EH, Grumbach K (2002). Improving primary care for patients with chronic illness: the chronic care model, Part 2. JAMA.

[3] Wagner EH, Austin BT, Von Korff M (1996). Organizing care for patients with chronic illness Milbank Q.

[4] Wagner EH (1998). Chronic disease management: What will it take to improve care for chronic illness?. Eff Clin Pract.

[5] Darves B (2005). Patient registries: A key step to quality improvement.

[6] Schmittdiel J, Bodenheimer T, Solomon NA, Gillies RR, Shortell SM (2005). Brief report: The prevalence and use of chronic disease registries in physician organizations. A national survey. J Gen Intern Med.

[7] Jamtvedt G, Young JM, Kristoffersen DT, Thomson O'Brien MA, Oxman AD (2003). Audit and feedback: Effects on professional practice and health care outcomes Cochrane Database. Syst Rev.

[8] Weingarten SR, Henning JM, Badamgarav E (2002). Interventions used in disease management programmes for patients with chronic illness–which ones work?. Meta-analysis of published reports. BMJ.

[9] Kenealy T, Arroll B, Petrie KJ (2005). Patients and computers as reminders to screen for diabetes in family practice. Randomized-controlled trial. J Gen Intern Med.

[10] Stroebel RJ, Scheitel SM, Fitz JS (2002). A randomized trial of three diabetes registry implementation strategies in a community internal medicine practice. Jt Comm J Qual Improv.

[11] Grant RW, Hamrick HE, Sullivan CM (2003). Impact of population management with direct physician feedback on care of patients with type 2 diabetes. Diabetes Care.

[12] Harris MF, Priddin D, Ruscoe W, Infante FA, O'Toole BI (2002). Quality of care provided by general practitioners using or not using division-based diabetes registers. Med J.

[13] Karter AJ, Parker MM, Moffet HH (2004). Missed appointments and poor glycemic control: An opportunity to identify high-risk diabetic patients. Med Care.

[14] Renders CM, Valk GD, Griffin S, Wagner EH, Eijk JT, Assendelft WJ (2001). Interventions to improve the management of diabetes mellitus in primary care, outpatient and community settings. Cochrane Database Syst Rev.

[15] (2007). Use of patient registries in US. primary care practices Am Fam Physician.

[16] Davis DA, Thomson MA, Oxman AD, Haynes RB (1995). Changing physician performance. A systematic review of the effect of continuing medical education strategies. JAMA.

[17] Lorig KR, Ritter P, Stewart AL (2001). Chronic disease self-management program: 2-year health status and health care utilization outcomes. Med Care.

[18] Ferguson JA, Weinberger M (1998). Case management programs in primary care. J Gen Intern Med.

[19] Rich MW, Beckham V, Wittenberg C, Leven CL, Freedland KE, Carney RM (1995). A multidisciplinary intervention to prevent the readmission of elderly patients with congestive heart failure. N Engl J Med.

[20] Al Rubeaan K A, Youssef A M, Subhani SN, Ahmad NA, Al Sharqawi AH, Ibrahim HM (2013). A Web-Based Interactive Diabetes Registry for Health Care Management and Planning in Saudi Arabia. J Med Internet Res.

[21] Geraghty EM, Balsbaugh T, Nuovo J, Sanjeev Tandon S (2010). Using Geographic Information Systems (GIS) to Assess Outcome Disparities in Patients with Type 2 Diabetes and Hyperlipidemia. JABFM.

[22] Curtis A, Kothari C, Paul R, Connors E (2013). Using GIS and Secondary Data to Target Diabetes-Related Public Health Efforts. Public Health Reports.

[23] Ministry of Health Eastern Regional Health Authority,Trinidad and Tobago.

[24] The CDEMS User Network (2010). CDEMS Basics.

[25] Diabetes Guidelines. A Desktop Guide to Type 2 Diabetes.

[26] Diagnosis and Classification of Diabetes Mellitus Diabetes Care 1997 20 1183-97 http://www.staff.ncl.ac.uk/philip.home/who_dmc.htm Accessed Feb, 2010 9203460 10.2337/diacare.20.7.1183

[27] The WHO classification for obesity.

[28] James PA, Oparil S, Carter BL (2014). 2014 Evidence-Based Guideline for the Management of High Blood Pressure in Adults: Report From the Panel Members Appointed to the Eighth Joint National Committee (JNC 8). JAMA.

[29] (2002). WHO Laboratory Diagnosis and monitoring of Diabetes Mellitus Manual. WHO Geneva.

[30] Pinhas-Hamiel O, Zeitler P (2005). The global spread of type 2 diabetes mellitus in children and adolescents. J Pediatr.

[31] MODY - It's Not Type 1 and Not Type 2, but Something Else. http://www.phlaunt.com/diabetes/14047009.php.

[32] Defronzo RA (2009). Banting Lecture. From the triumvirate to the ominous octet: a new paradigm for the treatment of type 2 diabetes mellitus. Diabetes.

[33] Fagot-Campagna A, Pettitt DJ, Engelgau MM, Burrows NR, Geiss LS, Valdez R, Beckles GL, Saaddine J, Gregg EW, Williamson DF, Narayan KM (2000). Type 2 diabetes among North American children and adolescents: an epidemiologic review and a public health perspective. J Pediatr.

[34] Poon King T, Henry MV, Rampersad F (1968). Prevalence and natural history of diabetes in Trinidad. The Lancet.

[35] Miller GJ Maude GH Beckles GL Incidence of HTN and Non- Insulin Dependent Diabetes Mellitus and associated risk factors in a rapidly developing Caribbean community: The St. James survey, Trinidad Journal of Epidemiology and Community Health 1996 50 497 -504 http://www.jstor.org/pss/25568320. 8944854 10.1136/jech.50.5.497 PMC1060339

[36] Chadee D, Seemungal T, Pinto Pereia LM, Chadee M, Maharaj R, Teelucksingh S (2013). Prevalence of self-reported diabetes, hypertension and heart disease in individuals seeking state funding in Trinidad and Tobago, West Indies. J Epid Glo Hea.

[37] American Diabetes Association (2015). Microvascular complications and foot care. Sec. 9. In Standards of Medical Care in Diabetes—2015. Diabetes Care.

[38] Boda P Availability and accessibility of diabetes clinics on Trinidad: An analysis using proximity tools in a GIS environment Health 2013 5 11B 35 -42

[39] (1990). Diabetes care and research in Europe: the Saint Vincent Declaration. Diabet Med.

[40] Mungrue K (2008). Are segments of the developing world competing in end-stage renal disease (ERSD)?. NDT Plus.

[41] The WHO classification for obesity http://apps.who.int/bmi/index.jsp?introPage=intro_3.html.

[42] Philip A, Ades MD, Patrick D, Savage MS (2010). The Obesity Paradox: Perception vs Knowledge. Mayo Clinic Proceedings February.

[43] Ford ES, Ajani UA, Croft JB, Critchley JA, Labarthe DR, Kottke TE (2007). Explaining the decrease in U.S. deaths from coronary disease, 1980-2000. N Engl J Med.

[44] Gregg EW, Cadwell BL, Cheng YJ, Cowie CC, Williams DE, Geiss L (2004). Trends in the prevalence and ratio of diagnosed to undiagnosed diabetes according to obesity levels in the US. Diabetes Care.

[45] Neil J, Stone (2014). 2013 ACC/AHA Guideline on the Treatment of Blood Cholesterol to Reduce Atherosclerotic Cardiovascular Risk in Adults: A Report of the American College of Cardiology/American Heart Association Task Force on Practice Guidelines.

[46] Mokdad AH, Ford ES, Bowman BA, Dietz WH, Vinicor F, Bales VS (2003). Prevalence of obesity, diabetes, and obesity - related health risk factors, 2001. JAMA.

[47] Konzem S, Pharm D (2002). Controlling Hypertension in Patients with Diabetes. Am Fam Physician.

[48] Sowers JR, Epstein M, Frohlich E D (2001). Diabetes, Hypertension, and Cardiovascular Disease. An Update Hypertension.

[49] Ricardo Padilla, Philip S, Mehler (2001). Journal of Women's Health & Gender-Based Medicine.

[50] Bodenheimer T, Wang MC, Rundall TG (2004). What are the facilitators and barriers in physician organizations' use of care management processes?. Jt Comm J Qual Saf.

